# Management of Symptomatic Elastofibroma Dorsi: A Case Report and Literature Review

**DOI:** 10.7759/cureus.29163

**Published:** 2022-09-14

**Authors:** Jamesa Fabien, Vijaykumar Patel, Megan Timpone

**Affiliations:** 1 Surgery, Ross University School of Medicine, Bridgetown, BRB; 2 General Surgery, Wellstar Atlanta Medical Center, East Point, USA; 3 General Surgery, Wellstar Kennestone Hospital, Atlanta, USA

**Keywords:** subscapular region, . surgical excision, benign tumors, fibroblastic tumor, elastofibroma dorsi

## Abstract

Elastofibroma dorsi (ED) is a rare tumor that most often occurs in the subscapular and infrascapular region between the thoracic wall, serratus anterior, and latissimus dorsi muscle. Based on a review of the literature, ED has been deemed an extremely rare entity. However, the incidence may be greater and is difficult to determine as the majority of ED being asymptomatic and therefore undiagnosed. Surgical excision is commonly performed when patients present with pain associated with ED. This being the case, it is important to evaluate the factors contributing to the pain seen in these patients and to evaluate the risks vs benefits of intervening in symptomatic ED patients who present for possible surgical intervention. We herein report a case of bilateral ED, situated in the upper back with only the right side being symptomatic in a 56-year-old male laborer. Due to pain in the right upper back, the patient underwent surgical removal of the ED. The postoperative course was uneventful and the patient had an excellent recovery. A review of the literature showed no correlation between pain on presentation and tumor size or location. Major complications of treating these patients include seroma or hematoma formation which according to the literature can be avoided using postoperative tube drainage and compressing bandages.

## Introduction

Elastofibroma dorsi (ED) is a rare tumor that most often occurs in the subscapular and infrascapular region between the thoracic wall, serratus anterior, and latissimus dorsi muscle [1]. ED has traditionally been considered a pseudotumor resulting from repetitive mechanical stress between the tip of the scapula and the thoracic wall leading to microtrauma and the subsequent overproduction of elastic tissue from stimulated fibroblasts. Thus, a relationship between manual/heavy activities and the development of ED, as in the case of our patient, has been investigated. Based on the existing literature, ED has been deemed an extremely rare condition, however, the incidence may be greater and is difficult to determine with the majority of ED being asymptomatic and therefore undiagnosed [2]. This being stated, it’s important to evaluate the risks vs benefits of intervening in symptomatic ED patients who present for possible surgical intervention. 

We herein report a case of bilateral ED, with only the right side being symptomatic, in a 56-year-old male laborer who underwent surgical excision on the right side of the upper back. We performed a literature review to investigate the potential factors that cause ED to be symptomatic and discuss potential surgical interventions given the risk for complications.

## Case presentation

A 56 y/o male presented with a painful right upper back mass which had gradually enlarged over the past two years. He had been experiencing a sharp non-radiating pain at the site of the mass, which he rated as seven out of ten on the pain scale. The patient had a previous history of a back injury that caused radiating back pain. He had no history of recent/new injury at the time of his presentation. The pain at the site of his mass was different from his chronic pain. The patient reported frequent manual labor at his job as a construction worker that continuously involved heavy lifting.

On physical exam, there was an 8 cm mass on the right upper back which was tender to palpation. The patient had no neurological signs. Range of motion (ROM) of upper extremities and shoulders remained intact bilaterally, with some mild discomfort on abduction and adduction of the right shoulder.

**Figure 1:**
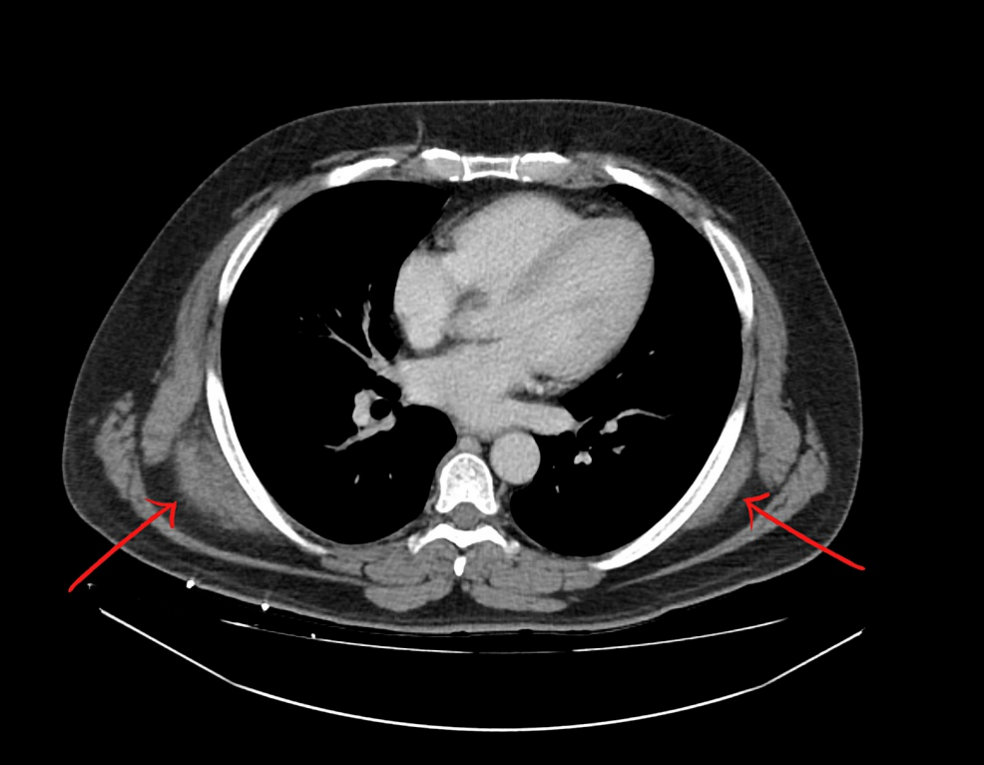
Bilateral Elastofibroma Dorsi Bilateral soft tissue masses (indicated by red arrows) present in the upper back immediately deep to the trapezius and latissimus dorsi muscles and posterior to the serratus musculature. The right mass measures 7.3 x 3.1 x 6.5 cm. The left mass measured 6.8 x 1.3 x 5.6 cm

CT scan of the chest with IV contrast showed bilateral soft tissue masses present in the upper back immediately deep to the trapezius and latissimus dorsi muscles and posterior to the serratus musculature (Figure 1). On the right, the mass measured 7.3 x 3.1 x 6.5 cm. On the left, the mass measured 6.8 x 1.3 x 5.6 cm. The CT scan findings were reported as consistent with ED

Given the patient’s persistent right-sided pain, lack of relief from over-the-counter medications, and the nature of his job, the patient requested surgical excision of the right-sided mass to relieve his pain and discomfort. 

Surgical excision was performed under general anesthesia. The mass was found located deep to the deep fascia and was noted to be intramuscular, measuring approximately 6 x 6 cm. Prior to closing the wound, meticulous hemostasis was obtained, and a Blake drain was placed deep in the intramuscular cavity to prevent seroma or hematoma formation The wound was closed in three layers. 

The specimen was sent for pathological evaluation. The pathology report stated that the lesion was found to be composed of fibrous tissue which showed a bland spindle cell component admixed as well as some adipose tissue. Trichrome stain showed extensive collagen deposition and elastic tissue stain demonstrated dense irregular clumps of elastic material. The specimen was strongly positive for vimentin, with smooth muscle actin, desmin, and s100 being negative. CD34 staining showed some positive cells and p53 staining showed some weak positivity. Based on these findings, pathology determined that these features were consistent with the clinical presentation of Elastofibroma, and no malignancy was identified.

Following discharge, the patient was seen on postoperative day six. The output of the patient’s Blake drain was consistently under 25mL per day since surgery and appeared serosanguinous. There were no signs or symptoms of post-operative infection. The Blake drain was removed during that visit. At the patient’s six-week follow-up, his surgical incision had healed well, and he had returned to his regular activities and work without restriction.

## Discussion

The PubMed Database was utilized to review previously published case reports and case series of ED focusing on tumor size vs symptoms and the number of cases with postoperative complications. Six individual case reports were included in our analysis, all of which reported pain on presentation with tumor size ranging from 5 x 5 cm to 13.5 x 8.7 cm (Table 1). There were no postoperative complications or recurrence reported in any of the case reports.

**Table 1:** Published case reports of Elastofibroma Dorsi

Published Case Reports, first author, year	Tumor size (cm)	Symptoms	Complications	Recurrence
Falidas et al., (2013) [1]	6.7 x 2.9	Pain	None	No
Go et al., (2014) [3]	10×6	Pain	None	No
Karrakchou et al., (2017) [4]	7.7 x 2.1 (right)	Pain	None	No
Karrakchou et al., (2017 [4]	8.3 x2.1 (left)	Asymptomatic	None	No
Sarici et al., (2014) [5]	5 x 5 (right)	Asymptomatic	None	No
Sarici et al., (2014) [5]	7 x 5 (left)	Pain	None	No
Pyne et al., (2002) [6]	5 x 5	Pain	None	No
Güzel et al., (2020) [7]	13.5 x 8.7	Pain	None	No

The rate of postoperative complications among the forty-two patients represented in the three case series analyzed was twenty-six percent (Table 2). There was no apparent correlation between size of tumor and pain on presentation as there were patients who presented with painful tumors as small as 3 cm, while other patients remained asymptomatic with tumors as large as 11 cm.

**Table 2:** Published Elastofibroma Dorsi Case Series n: sample size of study; NR: not reported

Published Series, First Author, Year	n	Symptomatic Patients (%)	Tumor diameter (cm)	Complication (%)
Nagano (2014) [8]	20	65	4.5 - 11	40
Tsikkinis (2014) [9]	6	NR	6.0-13.0	0
Karakurt (2014) [10]	16	100	3.0-13.0	19

Elastofibroma dorsi (ED), initially described as rare, was first reported and named by Jarvi et al in 1961, and was later classified as a benign fibroblastic/myofibroblastic tumor in 2000 by the World Health Organization [11]. Approximately 99% of elastofibromas are usually located in the soft tissue of the inferior angles of the scapulae, deep in the latissimus dorsi, serratus anterior and rhomboids, or lateral to the ribs and intercostal muscles [12]. It has traditionally been considered a pseudotumor resulting from repetitive mechanical stress between the tip of the scapula and the thoracic wall, leading to microtrauma and the subsequent overproduction of elastic tissue from stimulated fibroblasts [8]. However, the exact etiology remains unclear. 

Since forceful and repetitive movement of the shoulder girdle seems to be involved in ED pathogenesis, an investigation has been done looking at the relationship between manual\heavy activities and the development of ED which has shown a variable correlation of 15% to 95%, with male patients appearing to be more affected due to higher involvement in manual\heavy activities compared to females (71% and 30% respectively) [2]. In two different retrospective studies completed, bilateral ED appeared to be more commonly seen in up to 66% of patients [11]. A study completed by Haihua et al showed high-risk groups to include women aged between 50 and 70 years and men aged between 40 and 60 years [12]. As mentioned, ED was traditionally described as rare but is now thought to be more common given most small lesions remain asymptomatic with several studies showing CT-evaluation and autopsy series revealing a prevalence of 1.6% to 16.5% [2]. 

In cases of symptomatic ED, pain is the most common presenting chief complaint, with presentation ranging from mild to severely disabling pain. It has been assumed that the severity of the pain is dependent on the size and location of the tumor. However, based on our review, there was no clear correlation between tumor size and symptoms, with very small tumors presenting with pain and very large tumors remaining asymptomatic in some instances. Also, there was very little variation in the tumor location from our review with the majority of the tumors located in the subscapular region between the serratus and latissimus muscle. There is very little information from the existing literature on the factors contributing to painful ED. Further studies should be performed to evaluate the factors causing ED to become painful. [3-9].

The treatment of ED remains controversial. Given the benign nature of the lesion, surgery is reserved for symptomatic ED. The decision to excise an ED should be made jointly by the patient and surgeon. Those with significant symptoms should be offered an excision and be made aware of the risks of surgery [13]. The most common postoperative complications reported in the literature were seromas and hematomas. However, most patients did not have any post-operative complications. Additionally, the use of postoperative tube drainage and compressing bandages reduces the incidence of these complications. Furthermore, recurrence rates have been shown to be extremely low, with most patients reporting complete resolution of symptoms following surgical excision [10].

## Conclusions

Further investigations are needed to determine the factors contributing to pain in patients with ED since the size of ED does not appear to correlate with symptoms. Given that the rates of postoperative complications and recurrence are low, we recommend surgical excision be considered in all patients presenting with painful or symptomatic ED.
